# The Intracellular Bacteria *Chlamydia* Hijack Peroxisomes and Utilize Their Enzymatic Capacity to Produce Bacteria-Specific Phospholipids

**DOI:** 10.1371/journal.pone.0086196

**Published:** 2014-01-23

**Authors:** Gaelle Boncompain, Constanze Müller, Vannary Meas-Yedid, Philippe Schmitt-Kopplin, Paul B. Lazarow, Agathe Subtil

**Affiliations:** 1 Institut Pasteur, Unité de Biologie des Interactions Cellulaires, Paris, France; 2 CNRS URA 2582, Paris, France; 3 Department of BioGeoChemistry and Analytics, Institut für Ökologische Chemie, Helmholtz Zentrum München, Neuherberg, Germany; 4 Institut Pasteur, Unité Analyse d'images quantitative, Paris, France; University of Würzburg, Germany

## Abstract

*Chlamydia trachomatis* is an obligate intracellular pathogen responsible for loss of eyesight through trachoma and for millions of cases annually of sexually transmitted diseases. The bacteria develop within a membrane-bounded inclusion. They lack enzymes for several biosynthetic pathways, including those to make some phospholipids, and exploit their host to compensate. Three-dimensional fluorescence microscopy demonstrates that small organelles of the host, peroxisomes, are translocated into the *Chlamydia* inclusion and are found adjacent to the bacteria. In cells deficient for peroxisome biogenesis the bacteria are able to multiply and give rise to infectious progeny, demonstrating that peroxisomes are not essential for bacterial development *in vitro*. Mass spectrometry-based lipidomics reveal the presence in *C. trachomatis* of plasmalogens, ether phospholipids whose synthesis begins in peroxisomes and have never been described in aerobic bacteria before. Some of the bacterial plasmalogens are novel structures containing bacteria-specific odd-chain fatty acids; they are not made in uninfected cells nor in peroxisome-deficient cells. Their biosynthesis is thus accomplished by the metabolic collaboration of peroxisomes and bacteria.

## Introduction

*Chlamydia* are Gram-negative bacteria, which infect a wide range of hosts. They are obligate intracellular pathogens and multiply within mucosal epithelial cells. *Chlamydia trachomatis* is the aetiological agent of serious ocular and genital diseases having profound impacts on human health worldwide [1], [2]. Throughout evolution, chlamydiae have undergone extensive genome reduction, leading to the loss of several biosynthetic pathways. Regarding lipid synthesis, they possess the enzymes to synthesize some glycerophospholipids *de novo*, such as phosphatidylethanolamine and phosphatidylserine, but lack adequate genes to make others [3]. Elegant early studies demonstrated the transfer of typical eukaryotic host lipids to the bacteria, including phosphatidylcholine, sphingomyelin and cholesterol [4], [5], [6], [7]. Interestingly, host glycerophospholipids are not merely incorporated by the bacteria but some serve as precursors for bacteria-specific lipids: the *sn*-2 position fatty acid of the host phospholipids is removed and replaced with a *Chlamydia*-synthesized branched chain fatty acid with an odd number of carbons [6]. As a result, some bacterial phospholipids differ from those of the host cells, which have straight-chain fatty acids with an even number of carbons. Although it is clear that the bacteria acquire much of their lipid content from the host, the origin of these lipids and the transport pathways involved are not yet fully known [8]. The complete developmental cycle of these intracellular bacteria takes place within a membrane-bounded compartment, the inclusion [9]. Surprisingly, some of the host lipids reach the lumen of the inclusion in the form of lipid droplets (LD) [10], which are made up of a core of neutral lipids (triacylglycerol and sterol esters) surrounded by a phospholipid monolayer and a coat of proteins [11], [12]. The LD do not accumulate in the inclusion, suggesting that they are consumed [10], either for energy generation and/or as a source of fatty acids for lipid biosynthesis. Observations in mammalian cells [13] and in yeast [14], [15] demonstrate that LD are often found adjacent to peroxisomes with the membranes of the two organelles closely apposed/associated, which is believed to facilitate their metabolic collaboration. LD are thought to furnish substrates to some peroxisomal enzymes. Peroxisomes are small organelles present in virtually all eukaryotic cells [16]. They are surrounded by a single membrane and harbor enzymes engaged in an array of metabolic functions, including α- and β-oxidation of fatty acids, biosynthesis of ether lipids such as plasmalogens, metabolism of reactive oxygen species, synthesis of bile acids and metabolism of glyoxylate [17]. Since LD are imported into inclusions and peroxisomes are often associated with LD, we investigated whether peroxisomes might also be imported into inclusions and exploited by the bacteria.

## Results and Discussion

### Peroxisomes are translocated into the inclusion during infection

**Figure 1:**
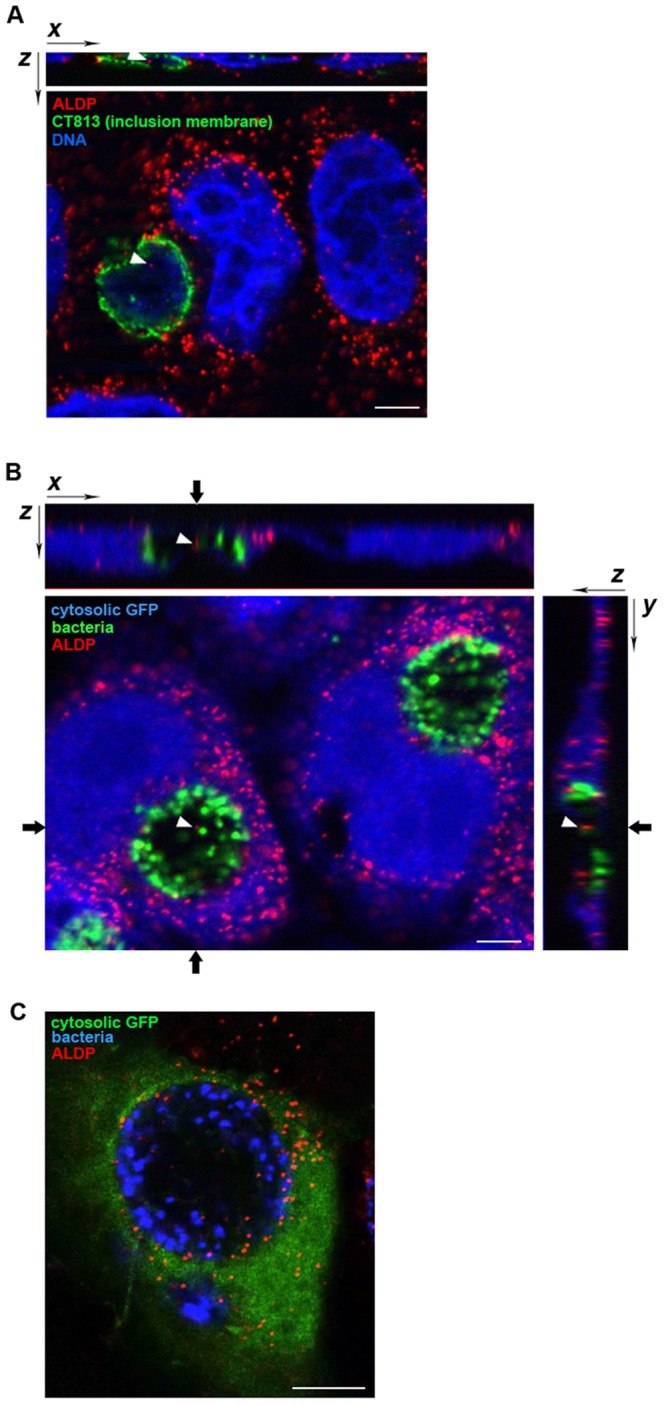
Peroxisomes are translocated into the *Chlamydia* inclusion during infection. A- HeLa cells were infected with *C. trachomatis* L2 for 20 h. The inclusion membrane was labeled with an anti-CT813 antibody (green), peroxisomes with an anti-ALDP antibody (red) and bacterial and nuclear DNA with Hoechst (Blue). A single ApoTome x-y section is shown in the central image. The z-x projection on the top shows the peroxisome indicated by a white arrowhead in the x-y image. Scale bar: 5 µm. B- HeLa cells were transfected with cytosolic GFP (shown in blue) to illuminate the entire cell *except* for the *Chlamydia* inclusion and were infected with *C. trachomatis* L2 for 20 h. Bacteria were labeled with an anti-Hsp60 antibody (green) and peroxisomes with an anti-ALDP (red). A single ApoTome x-y section is shown in the central image. z-x and z-y projections on the top and on the right side, respectively, are centered on the peroxisome indicated by a white arrowhead. Scale bar: 5 µm. C- One optical section from the stack of images shown in Movie S1. Cells were prepared as in Figure 1 B; the colors are different: bacteria are in blue, peroxisomes in red, GFP in green.

**Figure 2:**
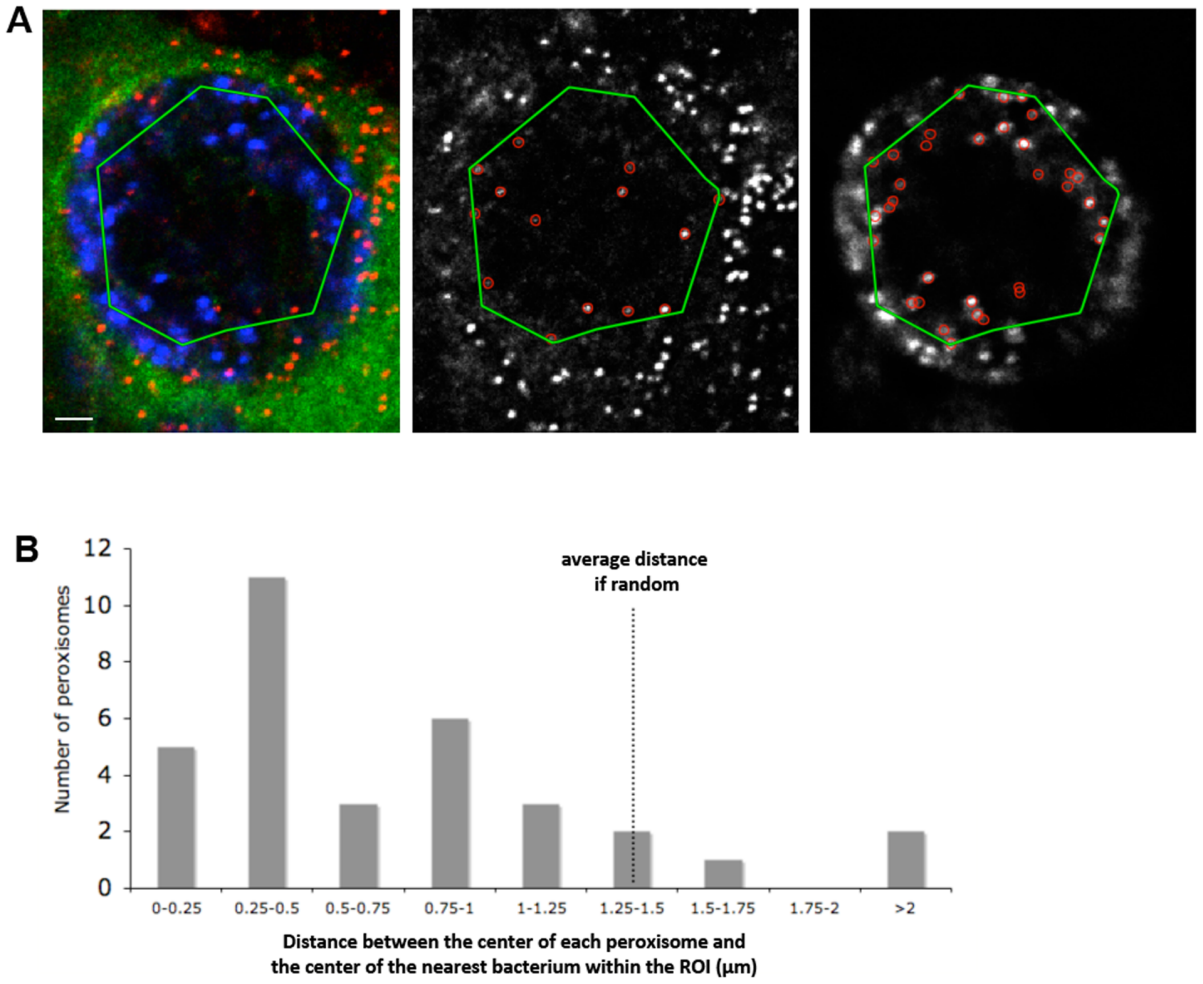
Peroxisomes are close to bacteria. A- Quantitative image analysis. A green polygon representing the Region Of Interest (ROI) was drawn over an optical section from the stack of images shown in Movie S1 (left image): bacteria are in blue, peroxisomes in red, GFP in green. Peroxisomes and bacteria detected within the ROI are circled in the middle and right images, respectively. Scale bar: 2 µm. B- Quantification of the distances between intra-inclusion peroxisomes and bacteria. The minimal distances between peroxisomes and bacteria within the ROI were calculated (from three different cells with respectively 6, 13 and 14 peroxisomes each, n = 33 peroxisomes in total) and the distribution of these distances is shown in the histogram. We calculated (see Methods) that a random distribution of bacteria and peroxisomes within the ROI would result in an average distance of 1.35 µm (p = 0.05, dotted line). The observed distribution is strongly shifted to the left and supports the hypothesis of a contact, or close proximity, between intra-inclusion peroxisomes and bacteria.

Two strategies were used to unambiguously determine the position of peroxisomes with respect to the inclusion volume by 3-dimensional fluorescence microscopy. First, the inclusion membrane was labeled with an antibody against the inclusion protein CT813 and optical sections of 0.7 µm were photographed through the entire thickness of the cells. We observed peroxisomes surrounding the inclusion and in many cases one or a few peroxisomes appeared to be inside. This was confirmed in the Z-projections of these fields: in the orthogonal slices, the peroxisomal labeling was within the luminal space of the CT813-labeled inclusion membrane (Figure 1A). Secondly, cells were transfected with GFP prior to infection so that the lumen of the inclusion appears as a black space in the green channel, as it is the only large volume inaccessible to GFP. Again, peroxisomes were observed inside the inclusion, often adjacent to anti-Hsp60 labeled bacteria (Figure 1B). Peroxisomes were observed in about half of the inclusion analyzed (n = 68). In this population, we scored on average 2.1 peroxisomes per inclusion (S.D. = 3.0). In some infected cells, many peroxisomes were found inside the inclusions (Figure 1C and Movie S1). Often they were very close to, or touching, a bacterium. The distances between peroxisomes and bacteria were quantified (Figure 2), which confirmed that peroxisomes are not randomly distributed in the inclusion but are mostly adjacent to, or within a short distance of, bacteria.

The observation that peroxisomes are actually translocated into the lumen of the inclusion is remarkable and confirms that the inclusion is capable of “ingesting” whole organelles [10]. We tried to image peroxisomes within inclusions by electron microscopy, but failed to do so. Intra-inclusion peroxisomes were regularly observed by immunofluorescence, but in most cells in low abundance, making this event difficult to catch at the ultrastructural level. The mechanism of capture and translocation of peroxisomes into the inclusion remains to be determined. They might enter the inclusion in association with LD [15], but the mechanism of LD import is also unknown. A 12-kDa chlamydial protein secreted into the host cytoplasm that targets LD, Lda3, might be implicated [18].

### Lack of peroxisomes causes smaller inclusions

**Figure 3:**
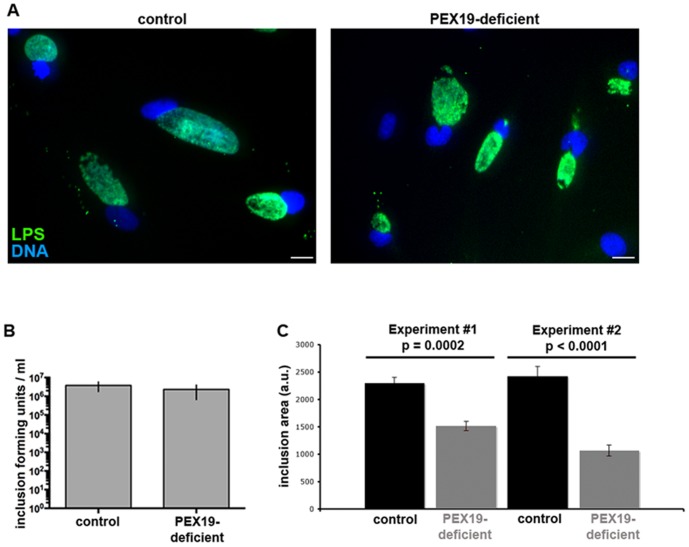
Peroxisomes are not essential for *C. trachomatis* infection and development. A- Control or PEX19-deficient fibroblasts were infected with *C. trachomatis* L2 for 24 h. Infected cells were fixed and labeled with an antibody against bacterial lipopolysaccharide (LPS) coupled to FITC (green) and DNA was stained with Hoechst (blue). Scale bar: 20 µm. B- The number of infection-forming units (IFU) was determined by titrating cell lysates at 48 hrs post infection on fresh HeLa cell monolayers. The average of three experiments is shown, error bars indicate standard deviation. C- Areas of inclusions. Results for two experiments are shown (Number of inclusions measured: experiment 1: control n = 111, PEX19-deficient n = 111; experiment 2: control n = 65, PEX19-deficient n = 76).

Defects in peroxisomal functions cause a variety of fatal inherited neurological diseases [19], [20]. Human fibroblasts lacking peroxisomes (due to a defective PEX19 gene, which is essential to assemble the peroxisome membrane [20]) and control human fibroblasts were infected with *C. trachomatis*. Inclusions formed in both cell types (Figure 3A), and there was no statistically significant difference in the production of infectious bacteria in the fibroblasts lacking peroxisomes compared to control fibroblasts (Figure 3B). The inclusions formed 24 hrs post infection in PEX19-deficient fibroblasts were 30 to 50% smaller compared to control (Figure 3C). Since the fibroblasts had been isolated from two different individuals, we cannot exclude the possibility that other differences between these two cell lines might account for this small growth defect measured in peroxisome-deficient cells. Therefore, while these results cleary show that peroxisomes are not essential for *Chlamydia* maturation in *in vitro* cell culture conditions, additional data will be needed to understand the role of peroxisomes in inclusion growth, in particular in tissues. Regarding this last question, it is useful to remember that mammalian cells are viable in culture without peroxisomes, but humans and mice die shortly after birth in the absence of peroxisomes.

### Many plasmalogens decrease in abundance during infection

The ability of peroxisomes to metabolize fatty acids and to participate in the synthesis of plasmalogens might be used by the bacteria. To test this hypothesis, we compared the lipid content of infected and uninfected human fibroblasts, and investigated the consequences of the absence of peroxisomes on the pattern of phospholipids. Phospholipids were extracted and separated in two dimensions by hydrophilic interaction liquid chromatography (HILIC), which separated the phosphatidylethanolamines from phosphatidylcholines (Figure S1B), followed by high performance reversed phase (RP) liquid chromatography (which separates by hydrophobicity) coupled to mass spectrometry (LC-MS) (Figure S1A). The data were combined in a matrix with the detected mass/charge (m/z), retention times and peak areas. Some of the extensive methods validation is shown in Figure S1. In addition to the wide variety of normal human phospholipids, in infected cells we identified phospholipids with fatty acids containing an odd number of carbons, which are characteristic of bacteria [21], including *Chlamydia* [6], [22], [23], and are absent from eukaryotic cells (Figure S1C).

**Figure 4:**
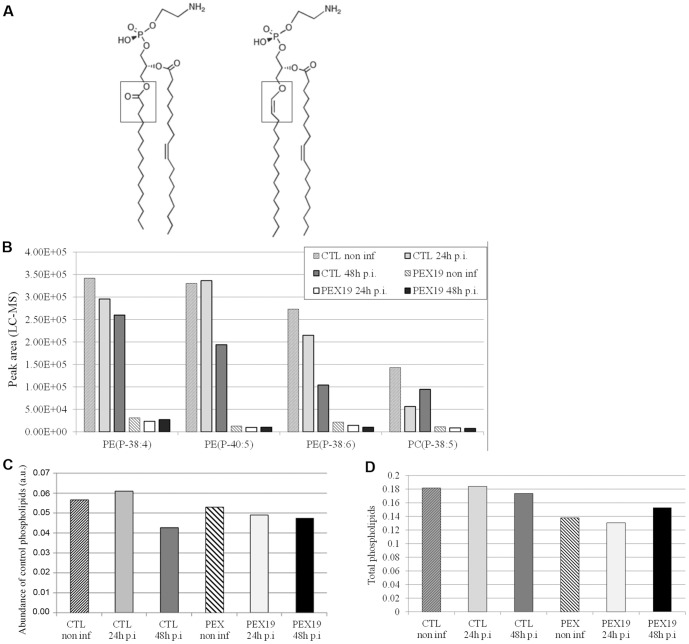
Many plasmalogens decrease upon infection. A- Structure of plasmalogens. Phosphatidylethanolamine (left) and the plasmalogen version of phosphatidylethanolamine (right) are depicted. The plasmalogen variant contains a long chain alcohol in place of a fatty acid and has a double bond between the first two carbons of the alcohol (1-O-alkenyl-2-acyl-*sn*-glycero-3-phosphoethanolamine; plasmenylethanolamine). B- Example of plasmalogens abundant in the non-infected control fibroblasts. Putative plasmalogens were identified based on their predicted formula as C_x_H_2x–2y_NO_7_P (where y = the number of double bonds in the fatty acid) after ICR-FT/MS analysis (mass error <100 parts/billion, MassTRIX [46]), and on their sensitivity to acid hydrolysis. Lipid species fulfilling these criteria were either missing or severely reduced in peroxisome-deficient cells (“PEX19”) compared to control fibroblasts (CTL), confirming that they are plasmalogens. Note that, globally, plasmalogen content decreased during infection, most notably at the later time point. Identification of plasmalogens was performed on samples from a single experiment. C- Abundance of control phospholipids. Phospholipids that gave the highest signal intensity in the ICR-FT/MS (corresponding to C42H82NO8P) were arbitrarily chosen as control. D- Total phospholipids detected with ICR-FT/MS. The intensities of all annotated phospholipids (mass error <1 ppm) were summed and normalized to the total sum of intensities in the sample. The data in C and D are representative of two independent experiments.

We paid particular attention to the plasmalogens because these phospholipids, which are minor components of most human cell membranes, but none-the-less essential to the normal development and health of mammals [20], [24], [25], [26], [27], require peroxisomal enzymes for the initial reactions of their biosynthesis [17], [28]. In cells in which peroxisome biogenesis is defective, such as the PEX-19 mutant cells described above, the biogenesis of plasmalogens is severely impaired. The plasmalogen variants of phosphatidylethanolamine (and other phospholipids) contain a long chain alcohol in place of a fatty acid and have a double bond between the first two carbons of the alcohol (1-O-alkenyl-2-acyl-*sn*-glycero-3-phosphoethanolamine; plasmenylethanolamine) (Figure 4A), which gives them a molecular composition of C_x_H_2x–2y_NO_7_P (where y is the number of double bonds, if any, in the fatty acid). The vinyl ether bond that links the alcohol of plasmalogens to the glycerophosphate backbone hydrolyzes readily under acidic conditions, whereas other phospholipid species are stable [29]. We detected known plasmalogens of normal human cells: their masses fitted a C_x_H_2x–2y_NO_7_P composition, they were acid-labile and they were greatly reduced in fibroblasts lacking peroxisomes (PEX19-defective), confirming that they are plasmalogens (Figure 4B). The most abundant plasmalogens in the samples, presumably of host origin, decreased (Figure 4B). In contrast, and in agreement with a previous report [6], we did not observe important changes in the global abundance of host cell phospholipids during infection.(Figure 4C).

### *Chlamydia* unexpectedly contain plasmalogens, with novel structures not found in the host cells

**Figure 5:**
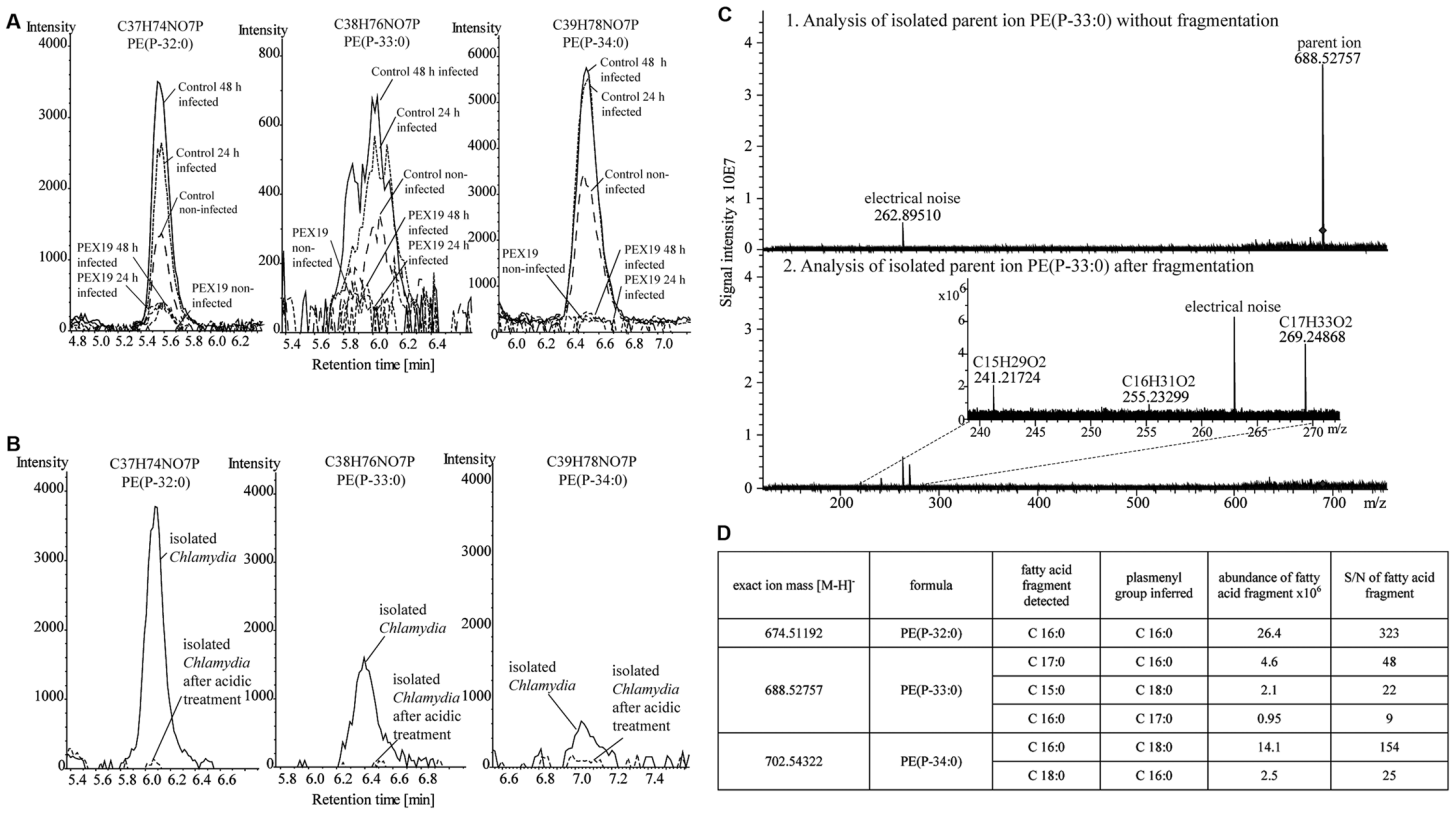
*C. trachomatis* contain bacteria-specific plasmalogens. A- Plasmalogens that increase in infected cells. Extracted ion chromatograms of plasmalogens of control and peroxisome-deficient (“PEX19”) fibroblasts, analyzed with RP LC-MS. The peaks of plasmalogens that increased upon 24 and 48 h of infection in control cells are shown. Notation, e.g. for PE(P-32:0): PE(P = plasmalogen version of phosphatidylethanolamine; 32:0 = 32 carbons in the combined side chains and 0 double bonds in the fatty acid. B- Plasmalogens in *C. trachomatis*. Phospholipids extracted from bacteria purified on a density gradient were analyzed as in B, with or without prior acid hydrolysis. The peaks with the same masses as in Panel B are shown. C- High resolution analysis and fragmentation of the plasmalogens of Panel C by SORI ICR/FT-MS^2^. The fragmentation pattern of PE(P-33:0) is shown as an example. D- Plasmalogen structures of *C. trachomatis*. Detected fatty acids and inferred structures for the *Chlamydia*-derived plasmalogens. S/N: signal to noise ratio.

Three LC-MS signals in the data matrix had the characteristics of plasmalogens and increased in abundance when *C. trachomatis* infected normal cells. As expected, these are minor constituents: they total less than 1% of all phospholipids. The two-dimensional LC-MS identified them as plasmenylethanolamines with a total of 32, 33 or 34 carbons in their side chains. These structures were confirmed at ultra high resolution (<100 parts/billion) by Ion Cyclotron Resonance Fourier Transform Mass Spectrometry (ICR-FT/MS) (Figure 5A). The same plasmalogens were found in purified *C. trachomatis* (Figure 5B). These data demonstrate that at least some of each of the plasmalogens that increases in infected cells is in the bacteria themselves.

The plasmalogens from the purified bacteria were fragmented by sustained off resonance irradiation (SORI) to determine the exact masses of the fatty acid substituents. The compound with 32 carbons in the combined side chains contained a 16-carbon fatty acid, implying by difference a 16-carbon plasmenyl group (Figure 5D). Fragmentation of the sample with 33 carbons in the combined side chains (Figure 5C) revealed a mix of 3 molecules, with fatty acids of 15 or 17 carbons, which are the odd-chain fatty acids synthesized by *C. trachomatis* [6], [22], [23], as well as one with a 16-carbon fatty acid (Figure 5C&D). Therefore, not only do *Chlamydia* contain host-derived plasmalogens, but also they modify them to produce *Chlamydia*-specific species. The sample with 34 carbons in the side chains contained 2 isomers with 16- or 18-carbon fatty acids, making a total of 6 plasmalogens in the *C. trachomatis*. From the determination of the fatty acid side chains, we could calculate the lengths of the alcohol side chains (Figure 5D). Most of them are the usual host-type fatty alcohols with an even number of carbons, but a small amount of a 17-carbon alcohol was also found; this likely has a bacterial origin too. Because the odd-chain fatty acids of *C. trachomatis* are reported to be all branched [6], [22], [23], presumably the odd-chain fatty acids that we find in the plasmalogens are branched, but this detail was not verified experimentally.

### Plasmalogens in *Chlamydia*: how and what for?

Intriguingly, this is the first example of an aerobic bacterium containing plasmalogens. Although plasmalogens are commonly found in strictly anaerobic bacteria and in animal species, they were believed to be entirely absent from aerobic bacteria and facultative aerobic bacteria, as well as from most fungi and plants [30]. Several related questions arise from this discovery: where and how are bacterial plasmalogens synthesized and what are they for? Our data give partial answers.

In animal cells, the first steps in the biosynthesis of plasmalogens are carried out by two enzymes found exclusively in peroxisomes: the dihydroxyacetone phosphate acyltransferase and the alkyl-dihydroxyacetone phosphate synthase. A third peroxisomal enzyme reduces the dihydroxyacetone to glycerol, producing a biosynthetic intermediate consisting of a long chain alcohol attached to glycerol phosphate, *i.e.* alkyl-glycerol-3-phosphate [17], [24], [28]. *C. trachomatis* can synthesize a normal phosphatidylethanolamine but its reduced genome does not encode the key enzymes of plasmalogen synthesis [3] (and the unknown open reading frames of *Chlamydia* are not predicted to do so). Fibroblasts without peroxisomes (PEX19-defective) do not begin to make plasmalogens upon infection (Figure 4B). Therefore, *Chlamydia* contain plasmalogens thanks to their exploitation of, and collaboration with, peroxisomes.

This process could occur by two non-exclusive scenarios. First, plasmalogens synthesized in the host cell cytoplasm may be imported first into the inclusion and subsequently into *Chlamydia*. Such a two-step mechanism operates for at least part of sphingomyelin and cholesterol transport, with the inclusion intercepting both vesicular [4], [7] and non-vesicular [31], [32] trafficking pathways. They could then be modified by *Chlamydia*, replacing the fatty acid at the sn-2 position with a 15- or 17-carbon bacterial fatty acid. Alternatively, the presence of peroxisomes within the inclusion suggests a more direct and novel collaboration between peroxisomes and *Chlamydia*. The intra-inclusion peroxisomes could produce the intermediate alkyl-glycerol-3-phosphate described above, which could be transferred to the bacterium to complete the plasmalogen synthesis with the bacterium's own enzymes of phospholipid synthesis. The fact that peroxisomes and bacteria are often adjacent (Figure 2) could facilitate such a transfer *via* membrane contact sites, as has been shown for lipid transfer between other membranes [33] including between bacterial and eukaryotic membranes [34].

Moreover, our data suggest a further intra-inclusion collaboration. One of the plasmalogens found in *Chlamydia* contains a 17-carbon long chain alcohol that could not be synthesized by the host cells. A 17-carbon fatty acid (linked to Coenzyme A) synthesized by *Chlamydia* could be reduced to the alcohol by the fatty acyl-CoA reductase that is conveniently located on the outer surface of an adjacent peroxisome [17], [25] and then incorporated into the biosynthetic intermediate. Thus peroxisomes and *Chlamydia* together could synthesize plasmalogens with either host or bacterial style fatty acids and/or alcohols. The lipid droplets within the inclusion are a ready source of the host fatty acids found in *C. trachomatis* phospholipids [10].

If some plasmalogens are imported from the host cell *via* the inclusion membrane, as has been reported for certain other host lipids, plasmalogens might remain in the inclusion membrane. The proportions of the several plasmalogens that increase upon infection are different in infected cells and purified bacteria (for instance PE(P-34:0) gives a stronger signal than PE(P-32:0) in infected cells but the reverse is observed in purified bacteria - see Figure 5A&B). This suggests that PE(P-34:0) is located in membrane(s) other than the bacterial membrane. It could well be the inclusion membrane, but testing this possibility was beyond the scope of the present study.

Another question raised by our results, what do *Chlamydia* use plasmalogens for, remains for future research. Plasmalogen functions include acting as antioxidants, modulating membrane dynamics, and serving as reservoirs of signaling molecules [24], [25], [27], [28], [30], [35]. The vinyl-ether bond of plasmalogens is readily susceptible to oxidants and can serve as a scavenger protecting other lipids from reactive oxygen species. It is possible that plasmalogens in *Chlamydia*'s membrane protect it against host-generated reactive oxygen [36]. In several cell types and tissues, plasmalogens serve as sources of arachidonic acid (liberated by a plasmalogen-specific phospholipase A2) that can be metabolized into other signaling molecules. *Chlamydia*-induced lipid metabolism is reported to contribute to prostaglandin E2 and interleukin-8 production during infection, but whether this involves plasmalogens was not investigated [37]. Our results show that plasmalogens are not essential to *Chlamydia* development. This is consistent with previous evidence that *Chlamydia* can adapt to reduced availability of other lipids they normally use. For example, phosphatidylcholine normally constitutes ∼30% of total lipids in infectious *Chlamydia*, but interfering with host phosphatidylcholine so as to decrease its content in bacteria by 40% had virtually no impact on the number of infectious progeny [5]. Similar observations were made concerning sphingomyelin.

### Other role(s) for peroxisome import into the inclusion

Plasmalogen acquisition is one benefit that bacteria get from hijacking peroxisomes into the inclusion. The ability of peroxisomes to perform β-oxidation of long chain fatty acids, at least to some extent [17], [38], might also be exploited by the bacteria, allowing them to utilize LD as a source of energy. This attractive possibility remains to be investigated experimentally. Another potential benefit is that host catalase, highly abundant in peroxisomes, might contribute to protecting *Chlamydia* against reactive oxygen produced during infection [36]. The *C. trachomatis* genome does not code for catalase, while environmental chlamydiae do; therefore hijacking peroxisomes might represent a way to compensate for catalase loss.

Several features, starting with their biphasic developmental cycle [9], make *Chlamydia* unique within prokaryotes. In this paper we report another remarkable novelty for this Gram-negative organism: the import of host peroxisomes into the inclusion and the use of this host organelle to shape their own lipid content and to produce bacteria-specific plasmalogens. Several plasmalogen species were identified within the complex mixture of host and bacterial phospholipids that increase in abundance during infection, in contrast to the bulk of plasmalogens, which decrease. The same plasmalogens that increase in infected cells were also found in the purified *C. trachomatis*, providing the first example of an aerobic bacterium containing plasmalogens. A great deal of research during the past 25 years has documented how diverse inherited diseases are caused by defects in peroxisomal enzymes [20] or by defective assembly of the organelle [19]. Diverse observations suggest a role for peroxisomes in viral infection [39]. The present results demonstrate that peroxisomes are imported into the lumen of the inclusion during *Chlamydia* infection and contribute to the synthesis of novel plasmalogens for this bacterium. This is the first case, to our knowledge, of a bacterium exploiting peroxisomes.

## Materials and Methods

### Cells, bacteria and antibodies

Human fibroblasts (gift of Dr. Ron Wanders, University of Amsterdam, described in [40]) and HeLa 229 cells (American Type Culture Collection) were grown in Dubelcco's modified Eagle's medium with GlutaMax (Invitrogen) supplemented with 10% fetal calf serum (Biowest) (complete medium). *C. trachomatis* LGV serovar L2 strain 434 was prepared as previously described [41]. Prior to lipid analysis bacteria were purified by density gradient centrifugation [42]. Antibody against Hsp60 was from from Pierce (#MA3-023), Hoechst 33342 was from Molecular Probes. Antibodies were kindly provided by Dr. G. Zhong (San Antonio, mouse anti-CT813), Pr. P. Aubourg and Dr. N. Cartier (Paris, rabbit anti-ALDP) respectively). Secondary antibodies were conjugated to Alexa488 or Alexa546 (Molecular Probes), to Cy3 or Cy5 (Amersham Biosciences).

### Immunofluorescence

Cells were fixed in 4% paraformaldehyde in 120 mM sucrose and phosphate buffered saline (PBS) for 20 min at room temperature and washed in PBS. Aldehydes were quenched with 50 mM NH_4_Cl for 5 min. The cells were washed in PBS and permeabilized with 0.05% saponin (Sigma) and 1 mg/ml of bovine serum albumin in PBS. Coverslips were mounted in Mowiol containing 0.5 µg/ml Hoechst 33342. Infected cells were fixed for 10 min (to avoid loss of CT813 signal at longer times). Optical sections (of 0.7 µm thickness) were acquired with an ApoTome microscope (Zeiss) equipped with a 63x objective and a Roper Scientific Coolsnap HQ camera; images were processed using AxioVision.

### Quantitative image analysis

Peroxisomes inside bacterial inclusions were observed with an epifluorescence microscope and optical slices were reconstructed by deconvolution. Image analysis was performed on one optical slice of the reconstructed stack. Peroxisomes and bacteria were identified using the Icy software ([43], http://icy.bioimageanalysis.org) by applying the spot extractor. Briefly, this method reconstructs the image from the thresholded wavelet bands such that the spots are denoised and enhanced. Three cells with a high number of peroxisomes per inclusion were chosen for the quantification. We then computed the distance between each peroxisome and each bacterium within the hand-drawn Region Of Interest (ROI), and extracted the minimal value. To determine whether the observed minimal distances were significantly shorter than what could be expected by chance, we built a null hypothesis model, in which the distribution of the distances between two independently randomly drawn spots in the cell is estimated by a kernel method [44]. We computed the distances of randomly drawn point-pairs for the number of peroxisomes and bacteria detected in each ROI. 3×10^6^ random distances were drawn, from which we could calculate that, for the 3 images analyzed, the average distance between a peroxisomes and the nearest bacterium would be 1.35 µm (±76 nm, S.E., p-value = 0.05) if these structures were randomly distributed.

### Lipid extraction and analysis

Lipid standards were purchased from Sigma Aldrich (St. Louis, USA) except for the plasmalogen standard, which was purchased from Avanti Polar Lipids, Inc. (Alabama, USA). Methanol, acetonitrile and water were used in LC-MS grade quality (CHROMASOLV®, Fluka® Analytical, Sigma-Aldrich). LC-MS grade n-propanol and ammonium formate were bought from BioSolve (Valkenswaard, Netherlands).

Fibroblasts (∼10^6^ for each sample, samples were normalized to identical cell numbers) were sonicated in 1 ml methanol for 15 s, centrifuged at 16,000×*g* for 15 min at 4°C, and the supernatant collected. A second methanol extraction was performed and the two supernatants were pooled. Lipids were similarly extracted from bacteria purified by density gradient centrifugation as described [42]. HILIC and RP LC-MS were performed on the crude lipid extracts with an ACQUITY UPLC® (Waters, Milford, USA) connected to an UHR QqToF mass spectrometer (maXis™, Bruker, Bremen, Germany). Molecules of interest were further analyzed at ultra-high resolution (<300,000 at m/z 300) and fragmented in negative electrospray ionization mode to identify the fatty acid side chains with an Ion Cyclotron Resonance Fourier Transform Mass Spectrometer (ICR-FT/MS, solariX™, Bruker Daltonics GmbH, Bremen, Germany) [45]. See Figure S1 and Movie S1 for further details and controls.

## Supporting Information

Figure S1 **Lipid analysis: workflow and methods validation.**
(DOC)

Movie S1 **HeLa cells were transfected with GFP (green) to illuminate the entire cell** ***except*** **for the** ***Chlamydia*** **inclusion and were infected with** ***C. trachomatis*** **L2 for 20 h.** Bacteria were labeled with anti-Hsp60 antibody (blue) and peroxisomes with anti-ALDP (red). A z-dimension series of images was acquired every 0.2 µm with Metamorph software and a 3D deconvolution microscope (DM-RXA2, Leica) equipped with a 100x Plan Apo NA 1.4 oil objective and a cooled charge-coupled device (CCD) camera (Roper CoolSnap HQ2). *Z*-dimension positioning was accomplished with a piezoelectric motor (LVDT, Physik Instrument). The images were then deconvoluted. Adjacent optical slices are displayed as adjacent frames in the movie.
(MOV)

## References

[1] Geisler WM (2010) Duration of Untreated, Uncomplicated Chlamydia trachomatis Genital Infection and Factors Associated with Chlamydia Resolution: A Review of Human Studies. J Infect Dis 201: S104–S113. 20470048 10.1086/652402

[2] Burton MJ, Mabey DC (2009) The global burden of trachoma: a review. PLoS Negl Trop Dis 3: e460. 19859534 10.1371/journal.pntd.0000460 PMC2761540

[3] Stephens RS, Kalman S, Lammel C, Fan J, Marathe R, et al (1998) Genome sequence of an obligate intracellular pathogen of humans: *Chlamydia trachomatis* . Science 282: 754–755. 9784136 10.1126/science.282.5389.754

[4] Hackstadt T, Scidmore MA, Rockey DD (1995) Lipid metabolism in *Chlamydia trachomatis*-infected cells: directed trafficking of Golgi-derived sphingolipids to the chlamydial inclusion. Proc Natl Acad Sci USA 92: 4877–4881. 7761416 10.1073/pnas.92.11.4877 PMC41810

[5] Hatch GM, McClarty G (1998) Phospholipid Composition of Purified *Chlamydia trachomatis* Mimics That of the Eucaryotic Host Cell. Infect Immun 66: 3727–3735. 9673255 10.1128/iai.66.8.3727-3735.1998 PMC108408

[6] Wylie JL, Hatch GM, McClarty G (1997) Host cell phospholipids are trafficked to and then modified by *Chlamydia trachomatis* . J Bacteriol 179: 7233–7242. 9393685 10.1128/jb.179.23.7233-7242.1997 PMC179671

[7] Carabeo RA, Mead DJ, Hackstadt T (2003) Golgi-dependent transport of cholesterol to the Chlamydia trachomatis inclusion. Proc Natl Acad Sci U S A 100: 6771–6776. 12743366 10.1073/pnas.1131289100 PMC164522

[8] Elwell CA, Engel JN (2012) Lipid acquisition by intracellular Chlamydiae. Cellular Microbiol 14: 1010–1018. 10.1111/j.1462-5822.2012.01794.x PMC3376245 22452394

[9] Scidmore MA (2011) Recent advances in Chlamydia subversion of host cytoskeletal and membrane trafficking pathways. Microbes Infect 13: 527–535. 21334451 10.1016/j.micinf.2011.02.001 PMC3092832

[10] Cocchiaro JL, Kumar Y, Fischer ER, Hackstadt T, Valdivia RH (2008) Cytoplasmic lipid droplets are translocated into the lumen of the Chlamydia trachomatis parasitophorous vacuole. Proc Natl Acad Sci U S A 105: 9379–9384. 18591669 10.1073/pnas.0712241105 PMC2453745

[11] Martin S, Parton RG (2006) Lipid droplets: a unified view of a dynamic organelle. Nat Rev Mol Cell Biol 7: 373–378. 16550215 10.1038/nrm1912

[12] Grillitsch K, Connerth M, Kofeler H, Arrey TN, Rietschel B, et al (2011) Lipid particles/droplets of the yeast Saccharomyces cerevisiae revisited: lipidome meets proteome. Biochim Biophys Acta 1811: 1165–1176. 21820081 10.1016/j.bbalip.2011.07.015 PMC3229976

[13] Schrader M (2001) Tubulo-reticular clusters of peroxisomes in living COS-7 cells: dynamic behavior and association with lipid droplets. J Histochem Cytochem 49: 1421–1429. 11668195 10.1177/002215540104901110

[14] Kohlwein SD, Veenhuis M, van der Klei IJ (2013) Lipid droplets and peroxisomes: key players in cellular lipid homeostasis or a matter of fat–store 'em up or burn 'em down. Genetics 193: 1–50. 23275493 10.1534/genetics.112.143362 PMC3527239

[15] Binns D, Januszewski T, Chen Y, Hill J, Markin VS, et al (2006) An intimate collaboration between peroxisomes and lipid bodies. J Cell Biol 173: 719–731. 16735577 10.1083/jcb.200511125 PMC2063889

[16] Islinger M, Grille S, Fahimi HD, Schrader M (2012) The peroxisome: an update on mysteries. Histochem Cell Biol 137: 547–574. 22415027 10.1007/s00418-012-0941-4

[17] Wanders RJ, Waterham HR (2006) Biochemistry of mammalian peroxisomes revisited. Annu Rev Biochem 75: 295–332. 16756494 10.1146/annurev.biochem.74.082803.133329

[18] Kumar Y, Cocchiaro J, Valdivia RH (2006) The obligate intracellular pathogen Chiamydia trachomatis targets host lipid droplets. Curr Biol 16: 1646–1651. 16920627 10.1016/j.cub.2006.06.060

[19] Steinberg SJ, Dodt G, Raymond GV, Braverman NE, Moser AB, et al (2006) Peroxisome biogenesis disorders. Biochim Biophys Acta 1763: 1733–1748. 17055079 10.1016/j.bbamcr.2006.09.010

[20] Wanders RJ, Waterham HR (2006) Peroxisomal disorders: the single peroxisomal enzyme deficiencies. Biochim Biophys Acta 1763: 1707–1720. 17055078 10.1016/j.bbamcr.2006.08.010

[21] Kaneda T (1991) Iso- and anteiso-fatty acids in bacteria: biosynthesis, function, and taxonomic significance. Microbiol Rev 55: 288–302. 1886522 10.1128/mr.55.2.288-302.1991 PMC372815

[22] Fan VS, Jenkin HM (1974) Lipid metabolism of monkey kidney cells (LLC-MK-2) infected with Chlamydia trachomatis strain lymphogranuloma venereum. Infect Immun 10: 464–470. 4214771 10.1128/iai.10.3.464-470.1974 PMC422976

[23] Larsson L, Jimenez J, Odham G, Westerdahl G, Mardh P-A (1982) Preliminary studies on cellular lipids of *Chlamydia trachomatis* using capillary gas chromatography. Chlamydial Infections. Amsterdam: Elsevier. pp. 37–40.

[24] Brites P, Waterham HR, Wanders RJ (2004) Functions and biosynthesis of plasmalogens in health and disease. Biochim Biophys Acta 1636: 219–231. 15164770 10.1016/j.bbalip.2003.12.010

[25] Braverman NE, Moser AB (2012) Functions of plasmalogen lipids in health and disease. Biochim Biophys Acta 1822: 1442–1452. 22627108 10.1016/j.bbadis.2012.05.008

[26] da Silva TF, Sousa VF, Malheiro AR, Brites P (2012) The importance of ether-phospholipids: A view from the perspective of mouse models. Biochim Biophys Acta 1822: 1501–1508. 22659211 10.1016/j.bbadis.2012.05.014

[27] Gorgas K, Teigler A, Komljenovic D, Just WW (2006) The ether lipid-deficient mouse: tracking down plasmalogen functions. Biochim Biophys Acta 1763: 1511–1526. 17027098 10.1016/j.bbamcr.2006.08.038

[28] Nagan N, Zoeller RA (2001) Plasmalogens: biosynthesis and functions. Prog Lipid Res 40: 199–229. 11275267 10.1016/s0163-7827(01)00003-0

[29] Murphy EJ, Stephens R, Jurkowitz-Alexander M, Horrocks LA (1993) Acidic hydrolysis of plasmalogens followed by high-performance liquid chromatography. Lipids 28: 565–568. 8355583 10.1007/BF02536090

[30] Goldfine H (2010) The appearance, disappearance and reappearance of plasmalogens in evolution. Prog Lipid Res 49: 493–498. 20637230 10.1016/j.plipres.2010.07.003

[31] Derre I, Swiss R, Agaisse H (2011) The lipid transfer protein CERT interacts with the Chlamydia inclusion protein IncD and participates to ER-Chlamydia inclusion membrane contact sites. PLoS Pathog 7: e1002092. 21731489 10.1371/journal.ppat.1002092 PMC3121800

[32] Elwell CA, Jiang S, Kim JH, Lee A, Wittmann T, et al (2011) Chlamydia trachomatis co-opts GBF1 and CERT to acquire host sphingomyelin for distinct roles during intracellular development. PLoS Pathog 7: e1002198. 21909260 10.1371/journal.ppat.1002198 PMC3164637

[33] Helle SC, Kanfer G, Kolar K, Lang A, Michel AH, et al (2013) Organization and function of membrane contact sites. Biochim Biophys Acta 10.1016/j.bbamcr.2013.01.028 23380708

[34] Crowley JT, Toledo AM, LaRocca TJ, Coleman JL, London E, et al (2013) Lipid Exchange between *Borrelia burgdorferi* and Host Cells. Plos Pathogens 9: e1003109. 23326230 10.1371/journal.ppat.1003109 PMC3542181

[35] Wallner S, Schmitz G (2011) Plasmalogens the neglected regulatory and scavenging lipid species. Chem Phys Lipids 164: 573–589. 21723266 10.1016/j.chemphyslip.2011.06.008

[36] Abdul-Sater AA, Said-Sadier N, Lam VM, Singh B, Pettengill MA, et al (2010) Enhancement of reactive oxygen species production and chlamydial infection by the mitochondrial Nod-like family member NLRX1. J Biol Chem 285: 41637–41645. 20959452 10.1074/jbc.M110.137885 PMC3009891

[37] Fukuda EY, Lad SP, Mikolon DP, Iacobelli-Martinez M, Li EG (2005) Activation of lipid metabolism contributes to interleukin-8 production during Chlamydia trachomatis infection of cervical epithelial cells. Infect Immun 73: 4017–4024. 15972489 10.1128/IAI.73.7.4017-4024.2005 PMC1168581

[38] Kondrup J, Lazarow PB (1985) Flux of palmitate through the peroxisomal and mitochondrial beta-oxidation systems in isolated rat hepatocytes. Biochim Biophys Acta 835: 147–153. 4005272 10.1016/0005-2760(85)90041-4

[39] Lazarow PB (2011) Viruses exploiting peroxisomes. Curr Opin Microbiol 14: 458–469. 21824805 10.1016/j.mib.2011.07.009

[40] Beemer FA, Wanders RJ, Schutgens RB (1990) VACTERL and hydrocephalus. Am J Med Genet 37: 425–426. 2260576 10.1002/ajmg.1320370325

[41] Boleti H, Benmerah A, Ojcius D, Cerf-Bensussan N, Dautry-Varsat A (1999) *Chlamydia* infection of epithelial cells expressing dynamin and Eps15 mutants: clathrin-independent entry into cells and dynamin-dependent productive growth. J Cell Sci 112: 1487–1496. 10212143 10.1242/jcs.112.10.1487

[42] Scidmore MA (2005) Cultivation and laboratory maintenance of *Chlamydia trachomatis* . Curr Protocols Microbiol 11A11.11–11A11.25. 10.1002/9780471729259.mc11a01s00 18770550

[43] de Chaumont F, Dallongeville S, Chenouard N, Herve N, Pop S, et al (2012) Icy: an open bioimage informatics platform for extended reproducible research. Nat Methods 9: 690–696. 22743774 10.1038/nmeth.2075

[44] Zhang B, Chenouard N, Olivo-Marin JC, Meas-Yedid V (2008) Statistical colocalization in biological imaging with false discovery control. IEEE Intern Symp on Biomedical Imaging, ISBI 2008, Paris, May 2008: 1327–1330.

[45] Kanawati B, von Saint Paul V, Herrmann C, Schaffner AR, Schmitt-Kopplin P (2011) Mass spectrometric stereoisomeric differentiation between alpha- and beta-ascorbic acid 2-O-glucosides. Experimental and density functional theory study. Rapid Commun Mass Spectrom 25: 806–814. 21337643 10.1002/rcm.4908

[46] Suhre K, Schmitt-Kopplin P (2008) MassTRIX: mass translator into pathways. Nucleic Acids Res 36: W481–484. 18442993 10.1093/nar/gkn194 PMC2447776

