# Supplementary material for: The Intracellular Bacteria Chlamydia Hijack Peroxisomes and Utilize Their Enzymatic Capacity to Produce Bacteria-Specific Phospholipids
Source: PLoS One. 2014 Jan 23;9(1):e86196. doi: 10.1371/journal.pone.0086196 (PMC3900481; doi:10.1371/journal.pone.0086196)
Supplement: Figure S1 — Lipid analysis: workflow and methods validation. (DOC) [file pone.0086196.s001.doc]

**Supplemental Information to** “The intracellular bacteria *Chlamydia* hijack peroxisomes and utilize their enzymatic capacity to produce bacteria-specific phospholipids ” by Boncompain et al.

- Fig. S1

- Supplemental Methods

- Supplemental References

- **Figure S1**- Lipid analysis: workflow and methods validation.

**A- Analytical workflow**.

The instrumental analysis of crude phospholipid extracts occurred in three phases:

1. combination of LC-MS methods with or without initial hydrolysis of vinyl ether bonds,

2. data analysis,

3. ICR-FT/MS and MS² for determination of exact masses and identification of lipid structures.

**B**- **Separation of phospholipid species.**

The crude lipid extracts have been analyzed by HILIC LC-MS and by RP LC-MS. These methods, which separate by hydrophilicity and hydrophobicity, respectively, give complementary information. The lipid extracts were also analyzed sequentially by the two techniques. This two-dimensional separation produces a high-resolution separation of the complex glycerophospholipid content of the samples. The procedure was tested with standards. Two diacylphosphoethanolamines (PE (18:1/18:1); PE (12:0/12:0)), two diacylphosphocholines (PC (18:0/18:1), PC (10:0/10:0)) and one plasmalogen (PC (P-18:0/18:1)) were mixed and analyzed with HILIC LC-MS as described in Supplemental Experimental Procedures. The retention times of the phosphatidylethanolamines were below 2.5 min and the retention times of the phosphatidylcholines were above 2.5 min, resulting in a good separation of these lipid classes, which were collected and afterwards re-injected for RP LC-MS separation.

Retention time (min)


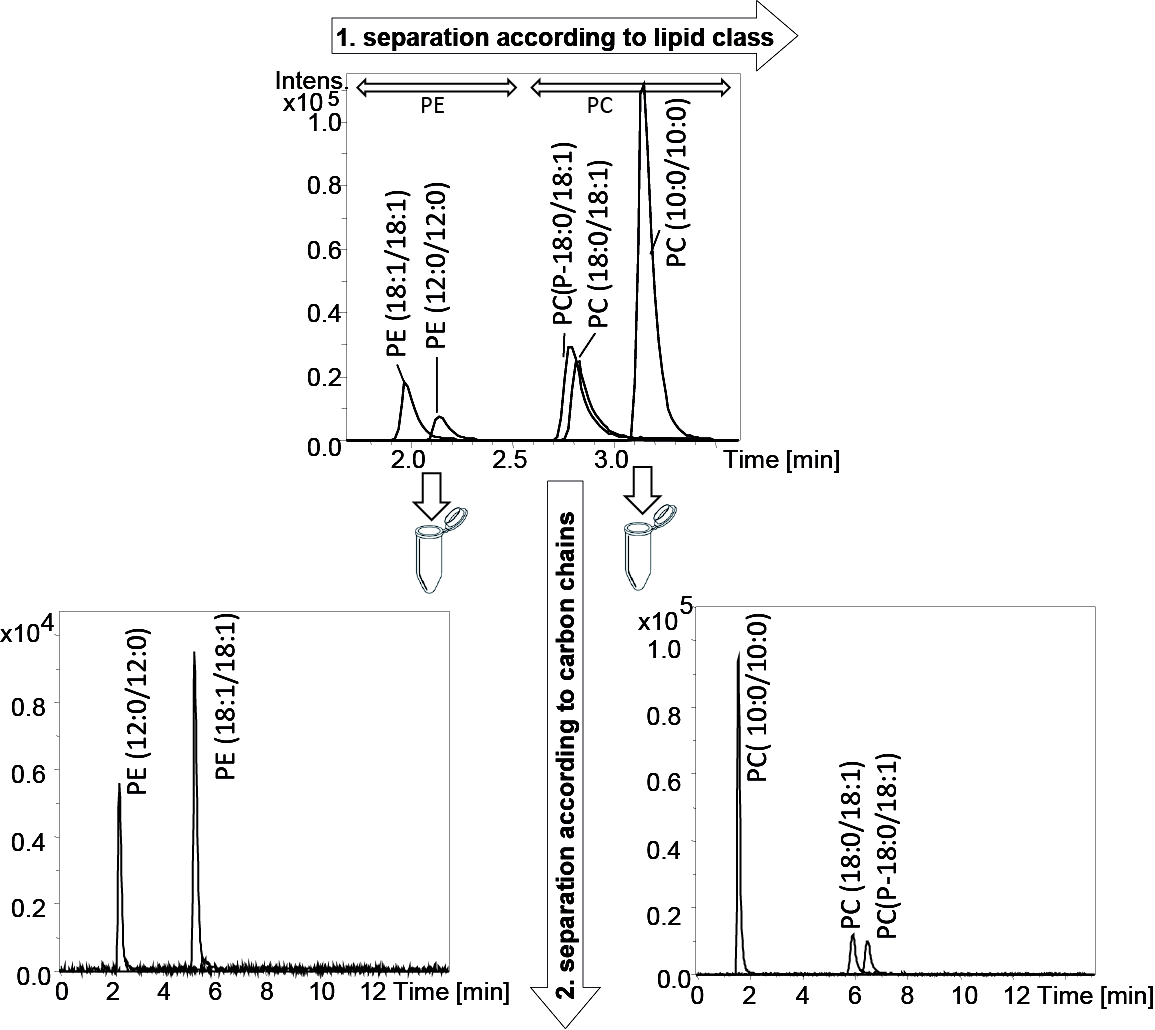


**C- Identification of bacteria specific fatty acids**

The data from the combination of HILIC LC-MS and RP LC-MS included phospholipids that were enriched in the infected samples compared to non-infected samples. We performed ICR-FT/MS² in negative electrospray mode on samples infected for 48 hrs to identify some of those lipids. In this procedure, the ions of interest (parent ions) are isolated and afterwards fragmented, resulting in informative patterns of product ions [1]. The TIC (total ion chromatograms, which gives all detected ions for one sample) showed very similar values in each samples, indicating that normalization between samples based on cell number was correct, and that the efficiency of lipid extraction was reproducible, Mass spectra consisting of 20 scans were acquired on parent ions and, after fragmentation, on product ions, which permitted a precise determination of the fatty acids present in these species. Several of them contained an odd number of carbons, and therefore correspond to branched chain fatty acids of bacterial origin. The peak areas (RP LC-MS) of some abundant phospholipids that contained such bacteria-specific fatty acids are shown below. They were equally abundant in peroxisome-deficient (PEX19) as in control (CTL) fibroblasts. In addition to these phospholipids containing odd chain fatty acids, we identified many other lipids only present, or greatly increased, in infected cells, most likely representing bacterial lipids. p.i.: post-infection.


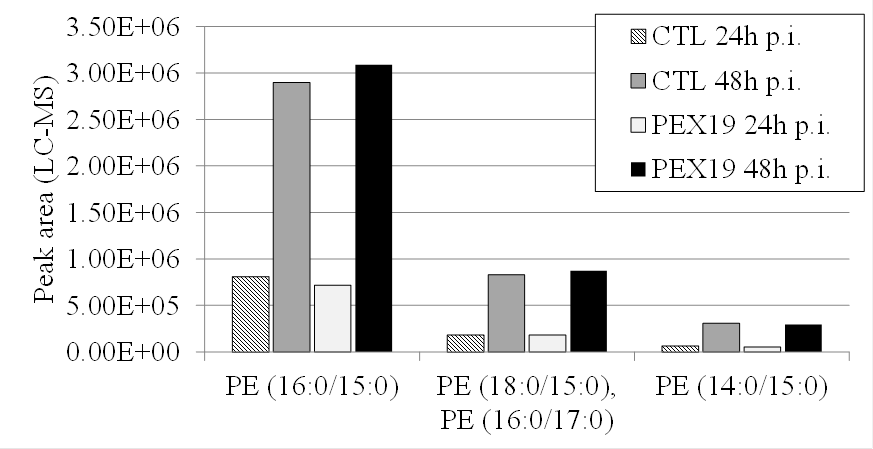


**Supplemental Methods**

*Lipid analysis*

An UHR QqToF instrument (maXis^TM^, Bruker, Bremen, Germany) was connected to an ACQUITY UPLC® (Waters, Milford, USA) for LC-MS analysis of crude phospholipid extracts. HILIC separation was performed on a Grace® VisionHTTM HILIC column (150 x 2.1 mm; 1.5 µm) using a 4 minute gradient from 6 % to 40 % solvent B followed by a 4 minute column equilibration time (solvent A 10 % water , 90 % acetonitrile, 5 mM ammonium formate; solvent B 5 % acetonitrile, 95 % water, 5 mM ammonium formate). The flow rate was optimized to 0.3 ml/min with a column temperature of 25 °C. RP analysis was done with an ACQUITY UPLC® HSS T3 column (150 x 1.0 mm, 1.7 µm) for second dimension separation. A 12 minute gradient from 60 % to 90 % solvent B was applied followed by a 1 minute plateau with 90 % solvent B (solvent A 60 % acetonitrile, 40 % water, 10 mM ammonium acetate; solvent B 90 % n-propanol, 10 % acetonitrile, 10 mM ammonium acetate). The flow rate was 0.11 ml/min. The column temperature was kept constant at 45 °C. ToF mass spectra were acquired in positive electrospray mode in order to detect within one run both phosphatidylcholines and phosphatidylethanolamines, which are the main classes of plasmalogens. Phosphatidycholines as zwitterionic species are mainly detectable in positive electrospray, while phosphatidylethanolamines are very well ionizable in both electrospray modes. Parameters of the ToF instrument were tuned for best resolution and accuracy in the mass range of approximately 500 - 900 Da. The data from the two LC-MS analyzes of were combined in a data matrix containing the detected m/z features, their retention times and peak areas, as well as their annotation. Annotation of the m/z features was carried out with MassTRIX applying a maximal mass error of 0.005 Da [2].

An Ion Cyclotron Resonance Fourier Transform Mass Spectrometer (ICR-FT/MS, solariX^TM^, Bruker Daltonics GmbH, Bremen, Germany) equipped with a 12 Tesla superconducting magnet was used for ultra-high resolution (300,000 at m/z 300) mass spectrometry [3]. Mass spectra were acquired in both positive and negative electrospray mode. Fragmentation experiments were done in negative electrospray ionization mode. The instrument was tuned to achieve best resolution and sensitivity in the m/z range of 500 - 900 Da in broad band detection mode. A time domain transient of 2 Megaword was applied. The ICR-FT/MS was calibrated with a 1 ppm arginine solution reaching a mass error below 100 ppb prior to the analyses. Chromatographic fractions of lipids of interest were injected with a flow rate of 2 µl/min to perform SORI fragmentation experiments for exact identification. The ionized plasmalogens were isolated inside the ICR cell with an isolation power of 20% and spectra of 80 scans were acquired. Thereafter the isolated ions were fragmented by application of 1.5% SORI power and spectra of the resulting product ions were acquired. MS on the fragmented lipids allowed identification of the fatty acids, and indirectly to infer the composition of the plasmenyl groups [1].

**Supplemental References**

1. Hsu FF, Turk J (2009) Electrospray ionization with low-energy collisionally activated dissociation tandem mass spectrometry of glycerophospholipids: mechanisms of fragmentation and structural characterization. J Chromatogr B Analyt Technol Biomed Life Sci 877: 2673-2695.

2. Suhre K, Schmitt-Kopplin P (2008) MassTRIX: mass translator into pathways. Nucleic Acids Res 36: W481-484.

3. Kanawati B, von Saint Paul V, Herrmann C, Schaffner AR, Schmitt-Kopplin P (2011) Mass spectrometric stereoisomeric differentiation between alpha- and beta-ascorbic acid 2-O-glucosides. Experimental and density functional theory study. Rapid Commun Mass Spectrom 25: 806-814.
